# Predicting Influenza A Virus Infection in the Lung from Hematological Data with Machine Learning

**DOI:** 10.1128/msystems.00459-22

**Published:** 2022-11-08

**Authors:** Suneet Singh Jhutty, Julia D. Boehme, Andreas Jeron, Julia Volckmar, Kristin Schultz, Jens Schreiber, Klaus Schughart, Kai Zhou, Jan Steinheimer, Horst Stöcker, Sabine Stegemann-Koniszewski, Dunja Bruder, Esteban A. Hernandez-Vargas

**Affiliations:** a Frankfurt Institute for Advanced Studies, Frankfurt am Main, Germany; b Faculty of Biological Sciences, Goethe University, Frankfurt am Main, Germany; c Immune Regulation Group, Helmholtz Centre for Infection Research, Braunschweig, Germany; d Infection Immunology Group, Institute of Medical Microbiology, Infection Control and Prevention, Health Campus Immunology, Infectiology and Inflammation, Otto-von-Guericke-University Magdeburg, Magdeburg, Germany; e Department of Infection Genetics, Helmholtz Centre for Infection Research, Braunschweig, Germany; f Department of Pneumology, Health Campus Immunology, Infectiology and Inflammation, Otto-von-Guericke University Magdeburg, Magdeburg, Germany; g Department of Microbiology, Immunology, and Biochemistry, University of Tennessee Health Science Center, Memphis, Tennessee, USA; h University of Veterinary Medicine Hannover, Hannover, Germany; i Institut für Theoretische Physik, Goethe Universität Frankfurt, Frankfurt am Main, Germany; j GSI Helmholtzzentrum für Schwerionenforschung GmbH, Darmstadt, Germany; k Department of Mathematics and Statistical Science, University of Idaho, Moscow, Idaho, USA; l Institute for Modeling Collaboration and Innovation, University of Idaho, Moscow, Idaho, USA; University of California San Diego

**Keywords:** influenza, machine learning, hematological parameters, cytokines, immune cells, biosystems, infectious disease

## Abstract

The tracking of pathogen burden and host responses with minimally invasive methods during respiratory infections is central for monitoring disease development and guiding treatment decisions. Utilizing a standardized murine model of respiratory influenza A virus (IAV) infection, we developed and tested different supervised machine learning models to predict viral burden and immune response markers, i.e., cytokines and leukocytes in the lung, from hematological data. We performed independently *in vivo* infection experiments to acquire extensive data for training and testing of the models. We show here that lung viral load, neutrophil counts, cytokines (such as gamma interferon [IFN-γ] and interleukin 6 [IL-6]), and other lung infection markers can be predicted from hematological data. Furthermore, feature analysis of the models showed that blood granulocytes and platelets play a crucial role in prediction and are highly involved in the immune response against IAV. The proposed *in silico* tools pave the path toward improved tracking and monitoring of influenza virus infections and possibly other respiratory infections based on minimally invasively obtained hematological parameters.

**IMPORTANCE** During the course of respiratory infections such as influenza, we do have a very limited view of immunological indicators to objectively and quantitatively evaluate the outcome of a host. Methods for monitoring immunological markers in a host’s lungs are invasive and expensive, and some of them are not feasible to perform. Using machine learning algorithms, we show for the first time that minimally invasively acquired hematological parameters can be used to infer lung viral burden, leukocytes, and cytokines following influenza virus infection in mice. The potential of the framework proposed here consists of a new qualitative vision of the disease processes in the lung compartment as a noninvasive tool.

## INTRODUCTION

Respiratory infections by influenza (flu) viruses cause 3 to 5 million cases of severe illness every year (1). Influenza A virus (IAV) is especially severe among high-risk groups like the elderly, infants, pregnant women, and immunocompromised people (2). In addition to its high prevalence in annual epidemics, IAV has led to high mortality during several pandemics, including the Spanish flu in 1918 and more recently the swine flu in 2009 (3, 4). Generally, the outcome of flu disease highly depends on viral factors as well as host immunity. Accordingly, a fatal course of infection can result from insufficient control of viral spread, hyperinflammation, and/or a secondary bacterial infection (5). Thus, tracking viral burden as well as host responses in the lungs is important for monitoring IAV pathogenesis and tailoring targeted therapies.

Methodologically, diagnosis and tracking of acute IAV infection can be performed by assessing viral antigens, nucleic acid, or infectious particles from upper or lower airway lavages, aspirates, or swabs. Likewise, monitoring of lower airway immune responses is accomplished by, e.g., quantification of inflammatory cytokines or leukocytes in bronchoalveolar lavage fluid (BALF). Next to several obvious disadvantages of these methods (low sensitivity, costly and time-consuming analyses, and/or high technical requirements), the biggest hurdle lies in the invasive sampling procedure, which poses a risk to the acutely infected patient (6). Accordingly, the development of noninvasive or minimally invasive approaches that allow observation of the molecular components of the immune system and viral dynamics during IAV infection remains to be explored.

Besides inducing acute inflammatory responses in the airways, influenza virus infection results in peripheral immune activation manifesting in altered blood cell composition (7), transcriptional signatures, and cytokine and chemokine levels in mice and humans (8–10). While longitudinal analyses revealed distinct molecular and/or cellular characteristics in peripheral blood of IAV-infected hosts, the suitability of each of these markers (and blood parameters in general) for predicting viral dynamics and immune cells in the lung compartment is still unknown. Next to the uncomplicated procedure of blood sample acquisition, there is a strong rationale to use hematology-derived information for surveillance of influenza virus infection. First, blood samples can be obtained at multiple time points following infection, which allows longitudinal analyses of influenza pathogenesis. Second, acquisition of hematological data can be performed within three to six hours using standardized, automated instruments that are routinely used in hospitals and many practices. This point-of-care testing (POCT) strategy might facilitate detection of illness at an early stage and enable rapid medical decisions.

Here, we propose for the first time a framework intertwining *in vivo* experiments and machine learning methods to forecast IAV infection parameters in the lung from blood sample data. To this end, we employed different machine-learning models for blood-lung mapping. Our primary computational approach was artificial neural networks, which represent a class of machine learning algorithms that use multiple layers of information processing for feature extraction and pattern analysis (11, 12). These methods have already been successfully applied in several biological fields, including the prediction of transcriptional enhancers (13), protein secondary structure (14), and the pathogenicity of genetic variants (15). Experimentally, we utilized an established mouse model of sublethal respiratory IAV infection (16, 17) and simultaneously assessed the kinetics of pathogen burden and lung inflammation, as well as systemic cellular changes following infection. Ultimately, several independent *in vivo* experiments were used to validate the applicability of the proposed framework.

## RESULTS

### Study design.

**FIG 1:**
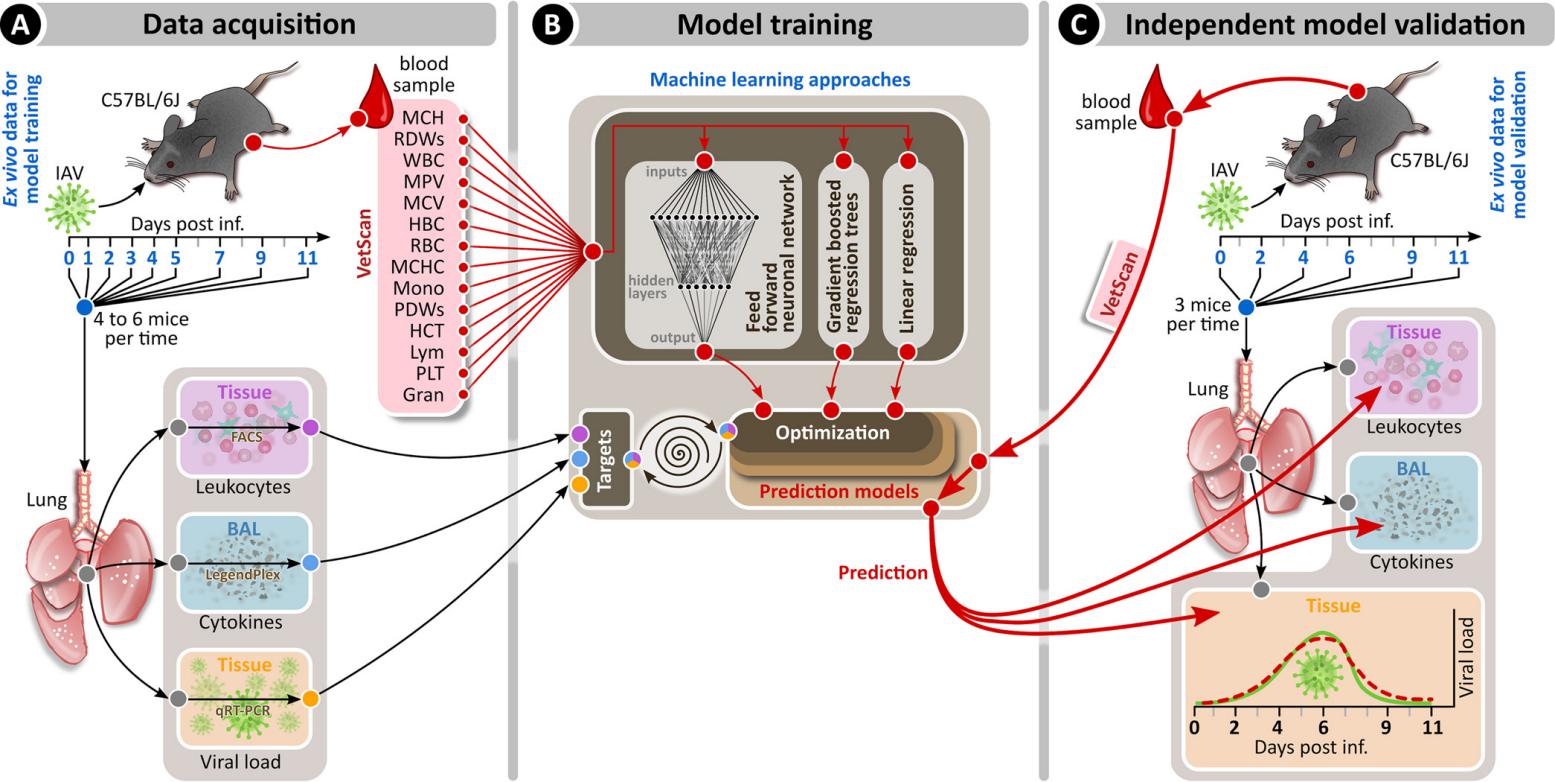
Experimental scheme for the machine learning approaches of the respiratory IAV infection. Mice were intranasally infected with a sublethal dose of IAV PR/8/34 on day 0 and sacrificed on the indicated days. Blood was collected for hematology analyses (Fig. S1 and S2), bronchoalveolar lavage was performed to analyze lung cytokines, and lung tissue samples were used to monitor either viral load (experiment 1 [Fig. S5]) or pulmonary leukocyte subsets (experiment 2 [Fig. S6 and S8]) (A). The hematological data from this initial set of experiments were used to build and train different machine learning models (B). Data from a separate experiment (Fig. S3 to S5, S7, and S9) were used for testing and evaluation of machine learning algorithms (C).

Our methodology consisted of three consecutive stages: *in vivo* experimental data acquisition, model training, and independent experimental model validation (Fig. 1). For the acquisition of experimental data, mice were infected with a sublethal dose of PR8 (H1N1) followed by the quantification of lung viral load, pulmonary innate and adaptive leukocyte subsets, pulmonary cytokine levels, and hematological parameters over a total period of 11 days. Blood and lung parameters were measured (Fig. 1) in two independent experiments; mice (*n* = 4 to 6 per time point) were sacrificed on days 1, 2, 3, 4, 5, 7, 9, and 11 postinfection (p.i.). These experimental data built the basis for model training using different machine learning approaches to identify the relationship between hematological and pulmonary parameters and to train and optimize the model accordingly. To validate the predictive value of our model, we performed two additional independent infection experiments with mice sacrificed on days 2, 4, 6, 9, and 11 p.i. and used the mathematical models to predict the lung viral burden, leukocyte composition, and cytokine levels based on experimental hematological parameters.

10.1128/msystems.00459-22.1 FIG S1 Results related to hematological parameters during IAV infection (first experiment, model setup and training). Download FIG S1, PDF file, 1.4 MB. Copyright © 2022 Jhutty et al. 2022 Jhutty et al. https://creativecommons.org/licenses/by/4.0/ This content is distributed under the terms of the Creative Commons Attribution 4.0 International license.

10.1128/msystems.00459-22.2 FIG S2 Results related to hematological parameters during IAV infection (second experiment, model setup and training). Download FIG S2, PDF file, 1.2 MB. Copyright © 2022 Jhutty et al. 2022 Jhutty et al. https://creativecommons.org/licenses/by/4.0/ This content is distributed under the terms of the Creative Commons Attribution 4.0 International license.

10.1128/msystems.00459-22.3 FIG S3 Results related to hematological parameters during IAV infection (third experiment, model validation). Download FIG S3, PDF file, 1.0 MB. Copyright © 2022 Jhutty et al. 2022 Jhutty et al. https://creativecommons.org/licenses/by/4.0/ This content is distributed under the terms of the Creative Commons Attribution 4.0 International license.

10.1128/msystems.00459-22.4 FIG S4 Results related to hematological parameters during IAV infection (fourth experiment, model validation). Download FIG S4, PDF file, 1.0 MB. Copyright © 2022 Jhutty et al. 2022 Jhutty et al. https://creativecommons.org/licenses/by/4.0/ This content is distributed under the terms of the Creative Commons Attribution 4.0 International license.

10.1128/msystems.00459-22.5 FIG S5 Results related to lung viral load during IAV infection. Download FIG S5, PDF file, 0.5 MB. Copyright © 2022 Jhutty et al. 2022 Jhutty et al. https://creativecommons.org/licenses/by/4.0/ This content is distributed under the terms of the Creative Commons Attribution 4.0 International license.

10.1128/msystems.00459-22.6 FIG S6 Results related to lung leukocytes during IAV infection (second experiment, model setup and training). Download FIG S6, PDF file, 1.1 MB. Copyright © 2022 Jhutty et al. 2022 Jhutty et al. https://creativecommons.org/licenses/by/4.0/ This content is distributed under the terms of the Creative Commons Attribution 4.0 International license.

10.1128/msystems.00459-22.7 FIG S7 Results related to lung leukocytes during IAV infection (fourth experiment, model validation). Download FIG S7, PDF file, 0.9 MB. Copyright © 2022 Jhutty et al. 2022 Jhutty et al. https://creativecommons.org/licenses/by/4.0/ This content is distributed under the terms of the Creative Commons Attribution 4.0 International license.

10.1128/msystems.00459-22.8 FIG S8 Results related to lung cytokines and chemokines during IAV infection (second experiment, model setup and training). Download FIG S8, PDF file, 1.5 MB. Copyright © 2022 Jhutty et al. 2022 Jhutty et al. https://creativecommons.org/licenses/by/4.0/ This content is distributed under the terms of the Creative Commons Attribution 4.0 International license.

10.1128/msystems.00459-22.9 FIG S9 Results related to lung cytokines and chemokines during IAV infection (fourth experiment, model validation). Download FIG S9, PDF file, 1.2 MB. Copyright © 2022 Jhutty et al. 2022 Jhutty et al. https://creativecommons.org/licenses/by/4.0/ This content is distributed under the terms of the Creative Commons Attribution 4.0 International license.

### Blood and lung data analysis.

**FIG 2:**
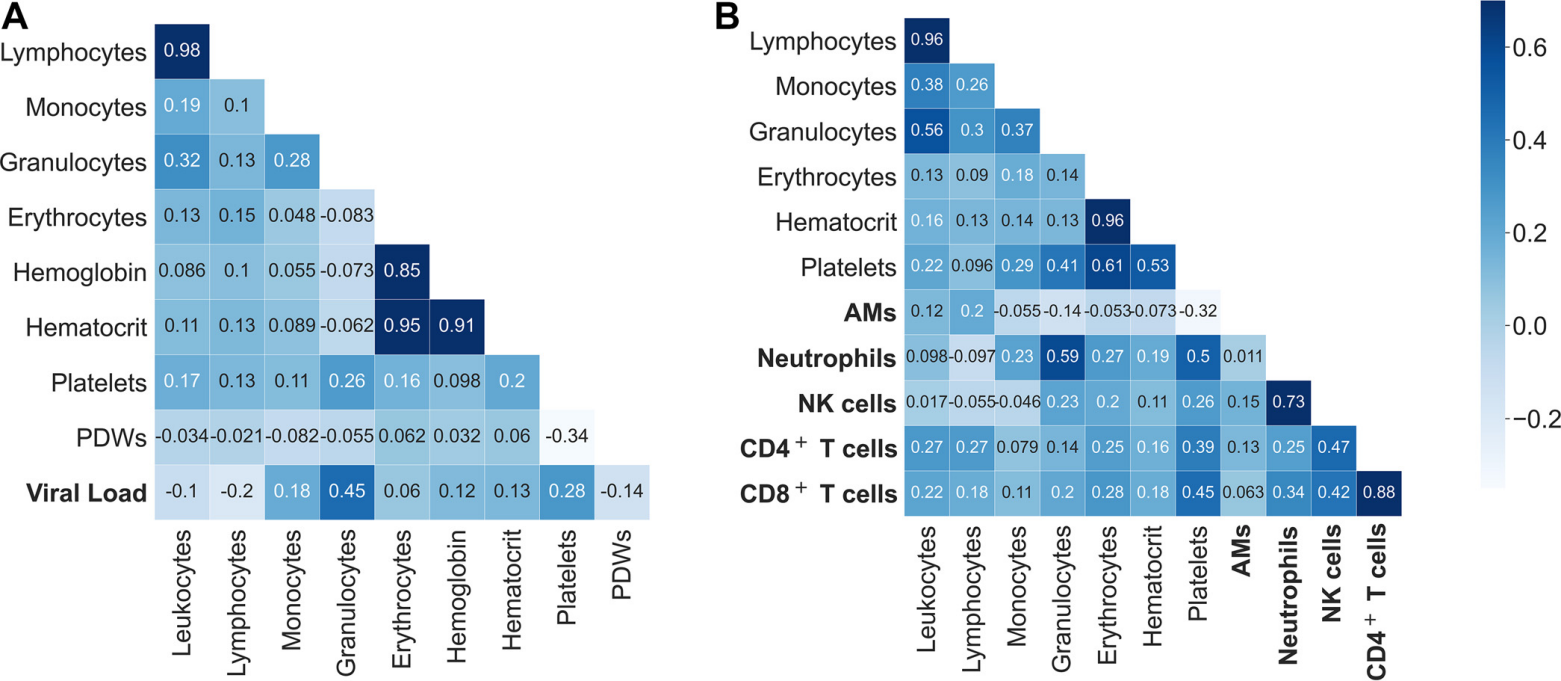
Selected correlations of blood cells, lung leukocytes, and lung viral load over the course of influenza virus infection. (A) Correlation of blood cells with lung viral load. (B) Correlation of blood cells with lung leukocytes. The matrices depict the respective Pearson correlation coefficients from the initial experiments used as training data for the machine learning models. We observed some strongly related clusters like erythrocytes, hemoglobin, and hematocrit or NK, CD4^+^ T, and CD8^+^ T cells. IAV-associated lung markers that were later predicted are shown in bold letters. All other parameters were provided to the algorithm to make the estimation.

To explore linear ties between selected hematological and pulmonary parameters, we conducted a correlation analysis on the longitudinal blood parameters based on the Pearson correlation coefficient (PCC) (Fig. 2). In accordance with common knowledge of immunology, we found a very strong correlation (PCC > 0.9) between blood leukocytes and lymphocytes, which can be attributed to the fact that lymphocytes constitute the largest leukocyte fraction in mice (18). Likewise, a strong correlation observed between hematocrit as well as hemoglobin and erythrocyte numbers (PCC > 0.8) can be attributed to the fact that hematocrit and hemoglobin are red blood cell-associated parameters (19).

**FIG 3:**
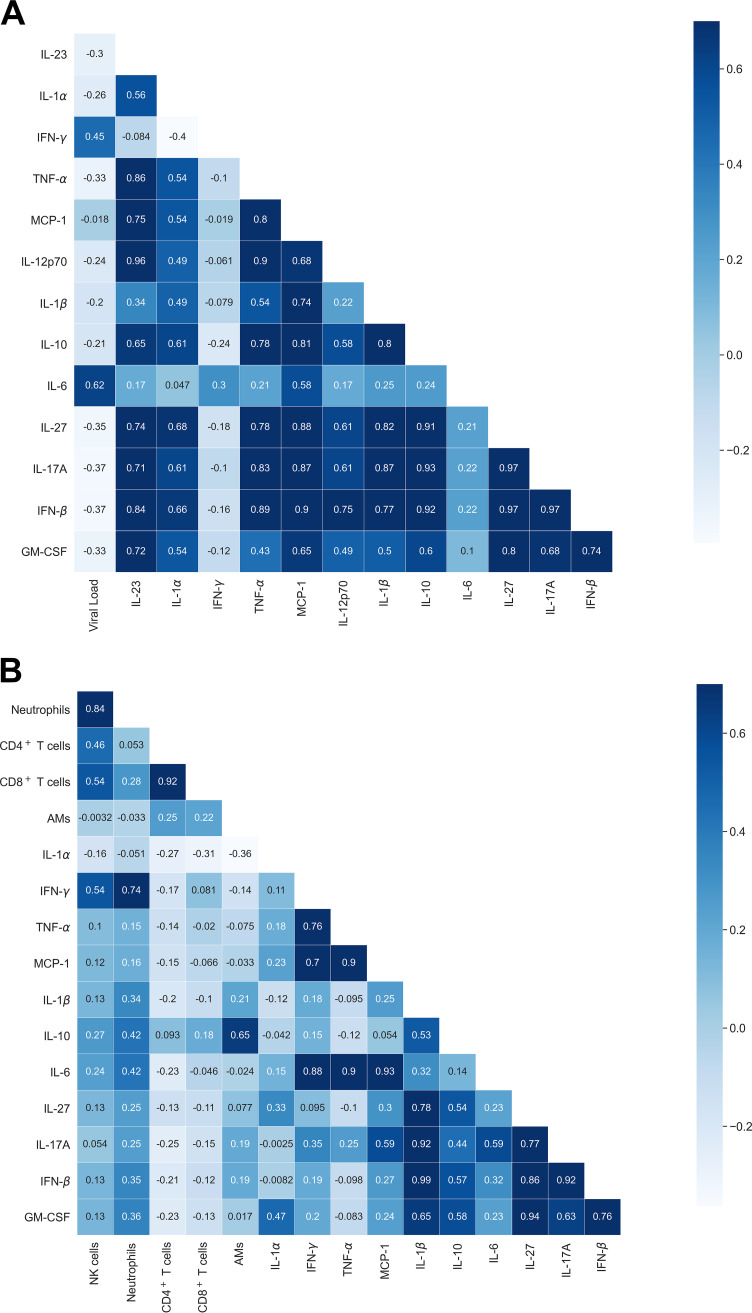
Correlations of cytokines from blood with lung viral load (A) and with lung leukocytes (B) over the course of influenza virus infection. The matrix depicts Pearson correlation coefficients from data of the fourth experiment.

As for cytokines in the blood compartment, we found that interleukin 6 (IL-6) showed the highest correlation with viral load in the lung compartment (PCC of about 0.62) (Fig. 3A). For lung leukocytes and cytokines in the blood, there were mild correlations, for instance, alveolar macrophages (AMs) and IL-10 (PCC around 0.65), as well as gamma interferon (IFN-γ) with both neutrophils (PCC of approximately 0.74) and natural killer (NK) cells (PCC of around 0.54) (Fig. 3B). Thus, the blood cytokines could be a relevant add-on in our approach; however, the multiparametric readout of blood cytokines is neither fast nor easy to perform and was therefore not our intended aim.

Strikingly, we did not observe any strong correlation between hematological parameters and cells in the lung compartment. However, there were strong correlations within cells of the lung compartment, such as CD4^+^ T cells and CD8^+^ T cells as well as neutrophils and NK cells. As a linear correlation does not include higher-order nonlinear temporal relations, we next employed machine learning algorithms and compared the obtained results with the linear regression model.

### Tracking IAV infection in the lungs from blood-derived parameters.

To predict influenza virus levels and immunological markers in the lungs from hematological parameters, we employed feedforward neural networks (FNN), gradient-boosted regression trees (GBRT), and a linear regression (LR) model. These algorithms considered 14 hematological parameters (Table 1) as features to predict the respective target lung variable. The main scores used for comparing and evaluating different machine learning algorithms were based on the average squared difference between the estimated values and the actual value (mean squared error [MSE]). The proportion of the variance in the dependent variable that is predictable from the independent variables was based on the *R*^2^ score. Overfitting was reduced with regularization techniques presented in Materials and Methods.

**TABLE 1:** Hematological parameters used to estimate the viral load in the lungs of mice^*a*^

Blood variable	Interpretation
Mean corpuscular hemoglobin	Avg amt of hemoglobin per red blood cell
Red blood cell distribution width	Degree of variation in size and shape of red blood cells
Leukocytes	Protect against infectious diseases and foreign bodies
Mean platelet vol	Avg size of platelets in blood
Mean corpuscular vol	Avg vol of red blood cells
Hemoglobin	Oxygen carrier in red blood cells
Erythrocytes	Oxygen transportation to the tissue
Mean corpuscular hemoglobin concn	Concn of hemoglobin in red blood cells per vol
Monocytes	Subtype of white blood cells
Platelet distribution width	Indicates variation in platelet size
Hematocrit	Vol percentage of red blood cells in blood
Lymphocytes	Subtype of white blood cells
Platelets	Blood component that helps stopping bleeding
Granulocytes	Subtype of white blood cells

a Blood variables are listed in order of their Pearson correlation with viral load (lowest coefficient, mean corpuscular hemoglobin; highest coefficient, granulocytes).

**FIG 4:**
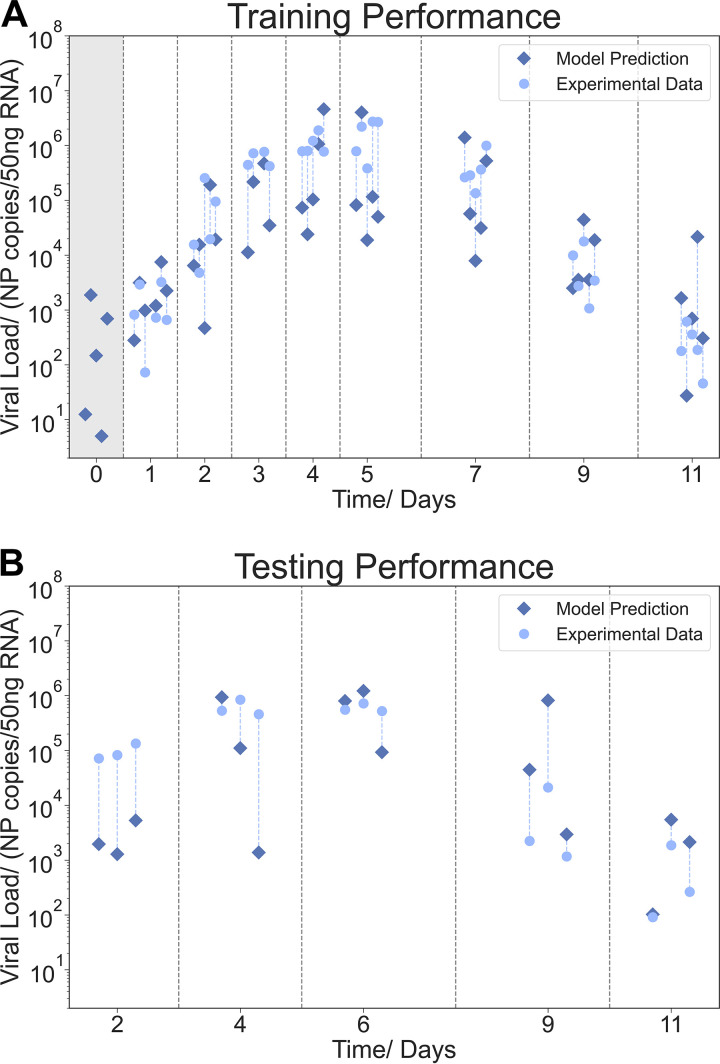
Mapping of the lung viral load from blood data. The plots show the training and testing performance of the neural network model. (A) Performance of the model on the training data. (B) Performance of the testing data obtained from a second experiment. Each circle represents one mouse, with its matching individual prediction indicated by a connected (blue dashed line) diamond. The vertical lines divide experimental days. Day 0 marks the control group (viral load below measurable threshold) and is highlighted in gray.

Figure 4A illustrates the best model prediction of viral levels in the lung, i.e., the feedforward neural network, based on experimental hematological data. We observed that the model can predict the general viral dynamics in the lungs (measured as copy numbers of nucleoprotein [NP] transcripts) over the experimental time frame of 11 days. Day 0 represents the control group. The prediction seemed to be most accurate around the peak of viral replication, i.e., days 4 and 5 p.i. Quantitatively, there was a high variation in the predictions. This was attributed to the variation found in our infection experiments (see Fig. S1 to S9 in the supplemental material), which is a common observation *in vitro* and *in vivo* viral infection experiments (20–25). While for some animals there was a large difference between the actual experimental data and the respective predictions in the testing experiments, overall, the qualitative performance on the testing set was good (Fig. 4B).

**FIG 5:**
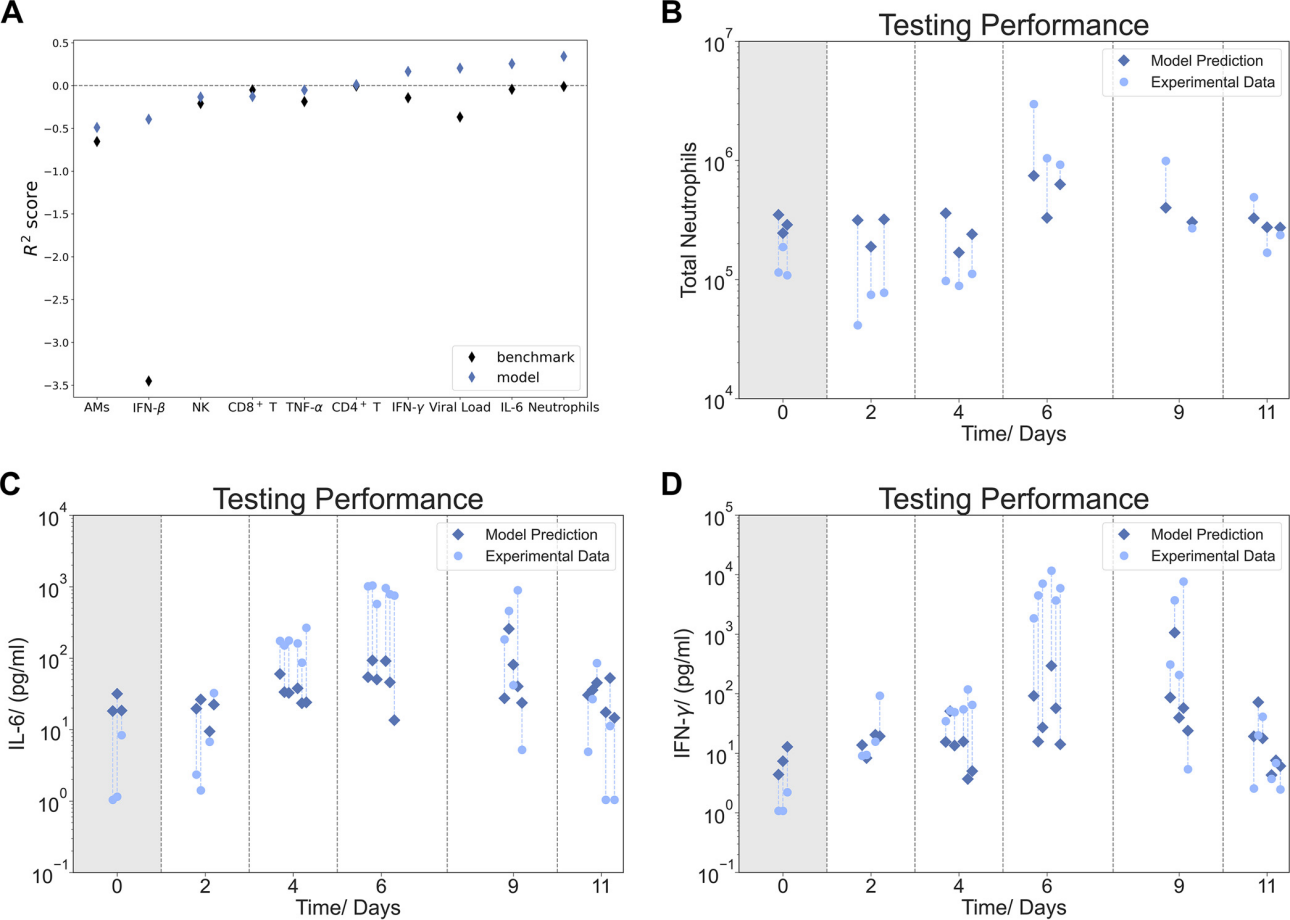
Summary of model predictions for various lung leukocytes and cytokines from hematological data. (A) *R*^2^ scores for different target variables. Blue diamonds show the *R*^2^ scores of the best-performing model. Black diamonds indicate the mean of the target variable obtained from the training data set and serve as a benchmark. Models that perform better than the benchmark and have a positive *R*^2^ score indicate the model can make successful predictions. (B to D) Mapping of neutrophils (B), IL-6 (C), and IFN-γ (D) from blood data. Each light blue circle represents one mouse, with its matching individual prediction indicated by a connected (blue dashed line) diamond. Gradient-boosted regression trees and linear regression with the aid of PCA worked best for these mappings. We observed that the quality of the predictions was dependent on the stage of the infection. Neutrophils were estimated more precisely in the advanced stage of infection, while for IL-6, more accurate estimations were yielded at the peak of the infection.

In addition to predicting the lung viral load, we tested several machine learning algorithms to predict target lung leukocytes and cytokines from the hematological parameters (Table 2). The comparison in Fig. 5A shows how the respective best model performed for different targets. As a benchmark, we used the mean for each target variable calculated from the training data. In almost all cases, the best model performed better than the benchmark. A positive *R*^2^ score demonstrates the explanatory power of the model. Predictions for lung IFN-γ, viral load, IL-6, and neutrophils were able to outperform the benchmark (Fig. 5).

**TABLE 2:** Best-performing model and respective scores for different targets from the lung milieu^*a*^

Target variable	Model	MSE	*R* ^2^
Viral load	FNN	7.53	0.18
Neutrophils	LR with PCA	0.98	0.25
IFN-γ	FNN	7.18	0.15
IL-6	GBRT	4.55	0.21

a Hematological data were used in all cases as input to the algorithms. For each target variable estimate, different machine learning models were tested.

Notably, the accuracy of the model predictions was dependent on the stage of the infection. For example, neutrophil numbers in the lungs and IL-6 and IFN-γ levels were better predicted in the later days of infection (Fig. 5B to D), while viral load was more accurately predicted at the peak of infection (Fig. 4B). This was also observed for other immune cells, such as CD4^+^ and CD8^+^ T cells. Table 2 presents a summary of the best models for predicting the different lung target parameters.

**FIG 6:**
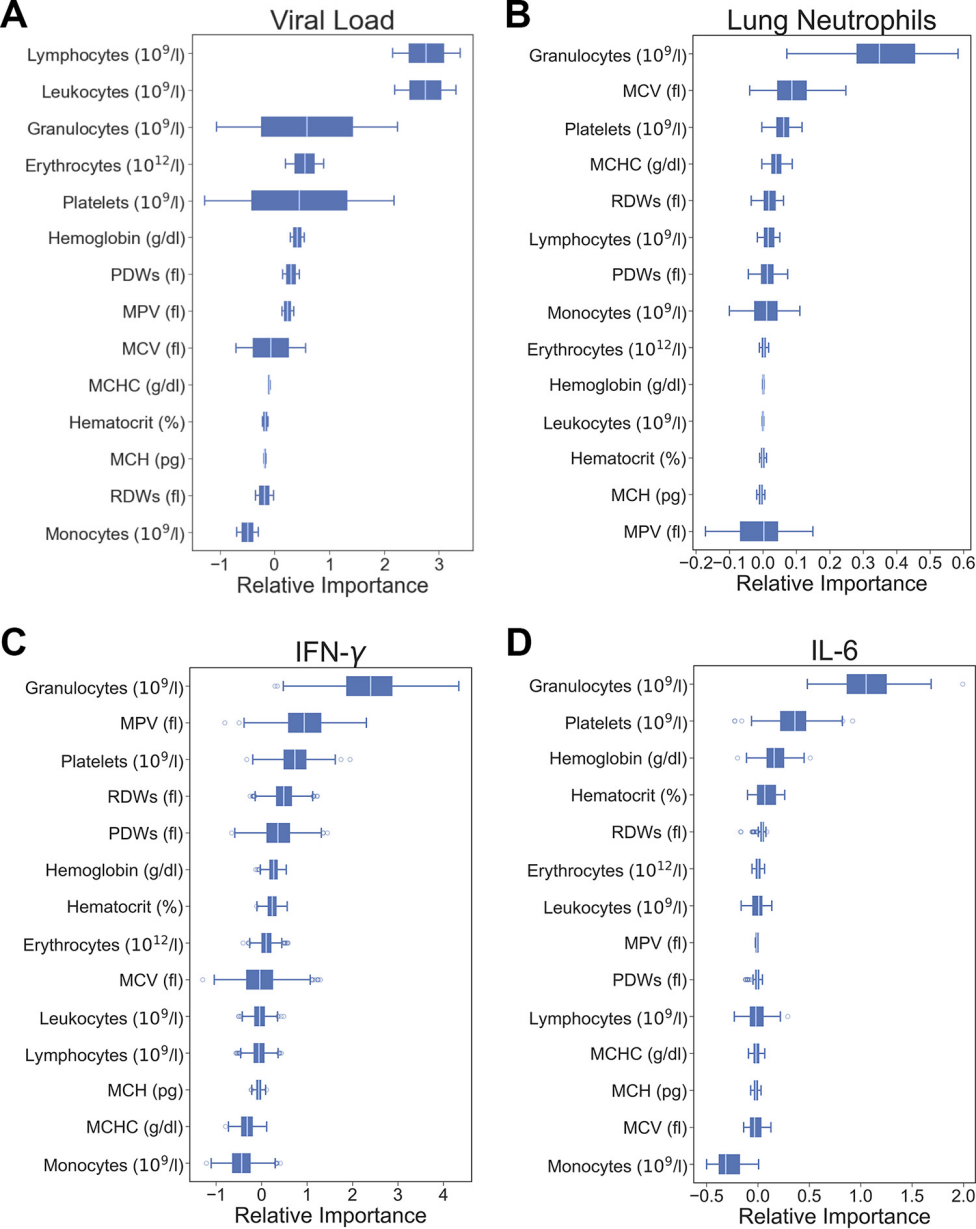
The permutation importance for the mapping of the viral load (A), neutrophils (B), IFN-γ (C), and IL-6 (D) is indicated. We calculated the permutation importance using the testing data.

To determine the role of each feature from the hematological data for the prediction of lung outcomes, we performed a feature importance analysis. For this, we calculated the permutation importance by swapping out features and evaluating the performance of our testing data. The results of the feature importance analysis are in Fig. 6. For example, from hematological parameters, granulocytes and platelets showed the greatest impact on the performance of the machine learning models for predicting the viral load, neutrophils, IFN-γ, and IL-6.

## DISCUSSION

Mathematical modeling of host immune responses has largely contributed to improving our understanding of the overall course of influenza virus infection (24, 26–34) as well as the personalization of therapies and vaccines (35–37). Mathematical models consist of systems of ordinary differential equations describing the viral dynamics within the host. However, computational tools for the diagnosis and tracking of respiratory diseases remain a public health challenge.

Here, we progress from the state of the art showing for the first time that minimally invasively acquired hematological parameters can be used to infer lung viral burden, some lung leukocyte populations, and some cytokines following IAV infection in mice. Nevertheless, despite standardized experimental procedures, our analysis showed a large variance in the computational predictions. These can be attributed to the relatively high variances of our experimental data due to biological or experimental variations. For instance, we found differences in some hematological parameters between the training and the testing experiments, which possibly explain the differences in performance between training and testing prediction of the lung viral load for day 2 postinfection.

Our study has several limitations, mainly with respect to the applicability of our approach to humans. The analysis was performed with only one inbred mouse strain. However, humans are genetically diverse and may be infected by influenza virus more than once. This can significantly influence the course of infection and immune response. To determine if genetic background may affect the observed results and our conclusions, future studies mimicking genetic diversity on the host population level are necessary. These would need to confirm the results in additional mouse inbred and possibly also outbred strains, allowing the controlled settings of animal experiments. Ultimately, our results propose a model that may be applied to human patients. For a transfer of our approach to humans, however, our model will furthermore have to be validated on existing data from controlled experimental human infections and clinical studies involving patients that display host heterogeneity with respect to genetics, health status, and the individual history of infections.

The potential of the framework proposed here consists of a new vision of the disease processes in the lung compartment. We show that the accumulation and decline of multiple cell types involved in the antiviral immune response in the lung can successfully be predicted with data derived from peripheral blood analyses. The boosted regression tree, with some modifications, provided the best results for many of the lung immune target cells. On the other hand, some target variables proved to be difficult to predict from hematological data. For instance, alveolar macrophage (AM) numbers could not be predicted with any of the tested algorithms and showed the worst score. This is likely the result of the weak correlations of AMs with the different blood cells analyzed. AMs also demonstrate quite different behavior than the other cells in terms of their abundance over the course of infection, as their number peaks rather late during the infection, i.e., when the virus is mostly cleared (38). This phenomenon is most likely the result of inflation of the alveolar macrophage pool by self-renewal (39) and/or monocyte recruitment processes (40). Regarding the less accurate predictions of cytokines within the airways from blood parameters, a possible explanation is that a large portion of these cytokines (especially during the early infection stage) originated from lung resident leukocytes as well as nonleukocytes. Therefore, their temporal quantity and composition are largely determined by local constituents of the lung’s immune cell response.

Our results show an active reaction chain between peripheral blood parameters and immune cells in the lungs of the mice following IAV infection. Interestingly, we found that peripheral blood platelets play an important role in predicting lung immune cell numbers in IAV infection. In line with our finding of increased numbers of platelets in the blood during the acute phase of IAV infection (see Fig. S1 in the supplemental material), platelet accumulation in the pulmonary capillaries is a hallmark of murine IAV H1N1 infection (41) and contributes to pathogenesis (42). Importantly, platelet-derived cytokines such as IL-1β can directly increase endothelial permeability and the expression of important vascular adhesion molecules (43, 44). In line with this, airway IL-1β levels were elevated during acute IAV infection (Fig. S8 and S9). Increased platelet-mediated transendothelial migration of CD4^+^ T cells, CD8^+^ T cells, NK cells, and neutrophils could thus be one conceivable mechanism contributing to the observed strong positive correlation between peripheral blood platelets and the aforementioned lung leukocyte subsets. This is relevant to the antiviral host response, as the CD4^+^ T cells are central in the activation and maturation of virus-specific CD8^+^ T cells (45), while neutrophils are required for proper NK cell maturation (46).

It should be noted that although the estimation is called “prediction” in the machine learning domain, and blood-derived data can be used in a practical way to “predict” the viral load or the number of certain lung immune cells or cytokines in IAV infection, we cannot establish a direction of causality in this case. In other words, we cannot state, e.g., that platelets are involved in raising the total amount of CD8^+^ T cells or if CD8^+^ T cells drive the increase in platelets. What we can learn from the correlations between variables is that CD8^+^ T cells, CD4^+^ T cells, NK cells, and neutrophils have their strongest positive correlation with platelets between the blood cells analyzed (Fig. 2). The feature importance analysis confirmed that platelets play the most important part in the estimation of lung CD4^+^ T and CD8^+^ T cells, followed by erythrocytes. However, the viral load inside the lungs has also a strong correlation with platelets but an even stronger one with granulocytes. The variable importance analysis suggests that platelets and granulocytes do not strongly contribute to the prediction, as can be seen in Fig. 6.

The weak predictive results obtained with the linear regression model signify that these relations have a high order of complexity. While contributing to the viral clearance, the innate immune system can also exacerbate the lung injury (47, 48). In this context, tissue injury can be a cause of platelet activation during influenza virus infection. The role of platelets in human influenza virus infection has been stressed in recent years (42, 49, 50). Thrombosis, controlled by the innate immune system, has been suggested to support immune defense (51).

Hematological parameters such as neutrophil, lymphocyte, and platelet counts, as well as the neutrophil-to-lymphocyte ratio (NLR), have contributed to diagnosing influenza virus infections (49). Thus, we also addressed if the use of the granulocyte-to-lymphocyte ratio (GLR) or the platelet-to-granulocyte ratio (PGR) improves importance in our model predictions. We compared the use of only the GLR or PGR with the use of only lymphocytes and granulocytes, as well using additional important IAV infection-associated peripheral blood parameters like erythrocytes and hemoglobin. Performances were evaluated with the MSE and *R*^2^ scores on the testing data set. We also calculated the corrected Akaike information criterion (AICc) during the training to take the complexity of the models into account. Table 3 shows that the model performance increased using GLR, while the use of PGR had only a minor effect. Also, adding erythrocytes and hemoglobin did not improve predictions.

**TABLE 3:** Comparison of different parameters as feature inputs for the viral load neural network model

Input features	MSE	*R* ^2^	AICc (training)
14 parameters	7.53	0.18	−0.22 ± 3.98
Replace lymphocytes, granulocytes with GLR	7.33	0.20	5.81 ± 5.51
Only lymphocytes, granulocytes	13.11	−0.42	18.37 ± 2.89
Only GLR, PGR	8.93	0.03	21.38 ± 2.11
Only erythrocytes, hemoglobin, GLR, PGR	9.00	0.03	19.68 ± 3.63

In summary, blood platelets, granulocytes, and erythrocytes play an important role in understanding the immune response to influenza virus infection and can be used in conjunction with other blood components for monitoring the lung viral load and lung immune cells in mice. Importantly, our results indicate that a reduced number of variables does not affect model/prediction accuracy. This can help to further reduce the hematology data needed for successful prediction. While recent efforts show evidence for the diagnosis of COVID-19 from the blood compartment (52), further clinical evidence will be needed to show the potential of how our procedure could be generalized to advance medical care.

## MATERIALS AND METHODS

### Experimental design.

Mice were intranasally infected with a sublethal dose of the mouse-adapted, strictly pneumotropic H1N1 IAV strain PR/8/34 and the lung viral burden, pulmonary innate and adaptive leukocyte subsets, pulmonary cytokine levels, and peripheral blood cell parameters were assessed for 11 days p.i. (Fig. 1).

Initial generation, training, and optimization of the computational algorithms were conducted using data from two independent *in vivo* infection experiments. Mice were randomly assigned to the respective experimental groups and were intranasally inoculated with either a sublethal dose of IAV or saline (control groups). Mice (*n* = 4 to 6/experimental group) were sacrificed on days 1, 2, 3, 4, 5, 7, 9, and 11 postinfection. Experimental readouts for the first experiment were hematological parameters and lung tissue viral load. In the second experiment, experimental readouts were hematological parameters, leukocytes in lung tissue, and airway cytokines.

For subsequent model validation, two additional, independent *in vivo* infection experiments were performed using the above-mentioned readouts. In these experiments, mice (*n* = 3/experimental group) were sacrificed at days 2, 4, 6, 9, and 11 postinfection. Hematological parameters used for model generation and evaluation are listed in Table 1.

### Mice.

For all experiments, female C57BL/6JOlaHsd mice (aged 10 to 12 weeks) from Envigo were used. All mice were housed in the animal facility at the Helmholtz Centre for Infection Research under specific-pathogen-free (SPF) conditions and in accordance with national and institutional guidelines. All the experiments were approved and conducted in accordance to the guidelines set by the local animal ethical bodies for the Helmholtz Centre for Infection Research (Niedersächsisches Landesamt für Verbraucherschutz und Lebensmittelsicherheit).

### Viral preparation and infection.

For viral infections, a mouse-adapted influenza A virus strain (A/Puerto Rico/8/34, H1N1) was utilized. The virus was produced in Madin-Darby canine kidney (MDCK) cells (53) and quantified by calculating the 50% tissue culture infectious dose (TCID_50_) as previously described (54). Mice were anesthetized by intraperitoneal injection of ketamine/xylazine and were infected with viral inoculum (0.31 TCID_50_ in 25 μL of phosphate-buffered saline [PBS]). Control animals received PBS only.

### Quantification of the lung viral load.

Lungs were perfused using PBS. RNA was extracted from whole-lung-tissue homogenates using the RNeasy Plus minikit (Qiagen). The absence of genomic DNA in RNA samples was initially confirmed by PCR using a *Taq* DNA-polymerase and primers for the housekeeping gene Rps9. Quantitative real-time reverse transcription (RT) PCR (qPCR) for detection of viral burden was performed using the SensiFAST SYBR No-ROX one-step kit and an influenza virus nucleoprotein (NP) plasmid standard. The sequences of the used primers were 5′-CTGGACGAGGGCAAGATGAAGC and 3′-TGACGTTGGCGGATGAGCACA (*Rps9*) and 5′-GAGGGGTGAGAATGGACGAAAAAC and 3′-CAGGCAGGCAGGCAGGACTT (*Np*).

### Hematology analysis.

Blood samples were obtained from the retrobulbar plexus and EDTA was added to prevent coagulation. Samples were analyzed using a VetScan HM5 machine (Abaxis).

### Cytokine detection.

Bronchoalveolar lavage (BAL) was performed with 1 mL of PBS, samples were spun down (420 × *g*, 10 min) and BAL fluid (BALF) supernatants were stored at −70°C until further analyses. Cytokine levels in BALF samples were quantified using the LEGENDplex mouse inflammation panel (BioLegend) according to the manufacturer’s protocol.

### Isolation of leukocytes from lung tissue.

Lungs were perfused using PBS, excised, and mechanically homogenized, and enzymatic digestion was performed in Iscove’s modified Dulbecco’s medium (IMDM) supplemented with 0.2 mg/mL of collagenase D (Roche), 0.01 mg/mL of DNase I (Sigma-Aldrich), and 5% fetal bovine serum at 37°C for 45 min. Digestion was stopped by the addition of EDTA, and the cell suspension was filtered through a 100-μm cell strainer and spun down (420 × *g*, 10 min). Erythrocyte lysis was performed using ammonium-chloride-potassium (ACK) buffer, and leukocytes were isolated by gradient centrifugation using Percoll solution (GE Healthcare). Lung tissue leukocytes were filtered again and antibody staining for flow cytometry was performed.

### Flow cytometry.

Lung tissue leukocyte samples were subjected to viability staining and blocking of Fc receptors using the LIVE/DEAD fixable blue dead cell stain kit (Life Technologies) and an anti-CD16/32 antibody (clone 93; BioLegend). Cells were then washed and incubated with a staining mix containing antibodies against the following murine antigens: Siglec-F (phycoerythrin [PE], clone E50-2440; BD), Ly6G (Alexa Fluor 700, clone 1A8; BD), CD11c (allophycocyanin [APC], clone N 418; BioLegend), CD11b (BV421, clone M1/70; BD), CD4 (APC-Fire750, clone GK1.5; BioLegend), CD8a (CyChrome, clone 53-6.7; BD), CD3ε (biotin, clone 145-2C11; BioLegend), and NK1.1 (fluorescein isothiocyanate [FITC], clone PK136; BioLegend). Secondary staining was performed using streptavidin-BV605 and streptavidin-BV650 (BioLegend). All reagents and antibodies had been titrated before the experiments for optimal staining results. Flow cytometry data were acquired using LSRII and LSR Fortessa instruments (BD). Data were analyzed using FlowJo software (BD).

### Data processing.

The data obtained from the hematological analysis consist of 20 different parameters. Some parameters are given in absolute values and percentages. We considered for these parameters only the absolute values, resulting in 14 different parameters (Table 1). For some mice, it was not possible to extract hematological data and/or target infection marker. These mice were removed for the mapping, although their data were used in the correlation analysis. Furthermore, if some values were lower than the measurable threshold, we used the threshold value. Computational algorithms were implemented in python using the Keras and sklearn libraries. Our study design yielded separate training and testing data sets. The testing data were obtained approximately 1 year after the training data. All laboratory conditions were kept as similar as possible. Using the data directly for training the algorithms led to poor results, and therefore, we used data preprocessing techniques. To conserve the nature of hematological parameter distributions, we used the minimum-maximum (min-max) scaling from the sklearn.preprocessing.MinMaxScaler class:

$$z=\frac{x-x_{\text{min}}}{x_{\text{max}}-x_{\text{min}}}$$

For the target infection markers like viral load, any logarithmic function worked well. For simplicity, we used log_10_.

### Machine learning models.

Different machine learning models were tested for the mapping, including feedforward neural networks (FNN), gradient-boosted regression trees (GBRT), linear regression (LR), support vector machines (SVM), and random forest regression (RFR). The hyperparameters of the models were estimated via grid search and adjusted via trial and error. FNN and GBRT were shown to be superior in most cases, and RFR was outperformed in every instance with one of the other algorithms.

In many cases, using principal-component analysis (PCA) before the mapping yielded improved performance. It was found that a dimensionality reduction to six input blood variables was often best. We used the class sklearn.decomposition.PCA for implementation. For the feedforward neural network, the keras library was used with a TensorFlow backend. We found that one hidden layer was sufficient most of the time and additional layers were not needed. The number of weights varied from 10 to 50. For regularization, the addition of dropout layers with a rate of 0.2 was helpful to prevent overfitting. As an activation function, we used a rectified linear unit (ReLU). We had one output that used a linear activation function. This was necessary to map the whole range of possible outcome values. We used the Adam optimizer and minimized the mean squared error to find the optimal fit. The weights were initialized according to (55). Following common practice in literature, we used for the training of the neural network a validation set of 10% of the whole training data.

The GBRT, LR, SVM, and RFR algorithms were taken from the python library sklearn. The hyperparameters of GBRT and RFR models were searched over a grid from 10 to 2,000 estimators, a learning rate from 0.001 to 0.09, and a maximum depth of 2 to 14. The least-square regression was used for optimization. The kernels used for SVM were “*linear*,” “*poly*,” “*rbf*,” “*sigmoid*,” and “*precomputed*.”

To determine which variables were the most important in our model predictions, we calculated the permutation importance using the *sklearn.inspection.permutation_importance* implementation. For this, we took our best model and trained it on the training data set. After the trained model was evaluated on the hold-out testing data set with the mean squared error as the metric, a feature column was permuted and the metric was evaluated again. This procedure was repeated 100 times, and the permutation importance was given by the difference between the baseline metric and the metric from permutated feature columns.

### Data availability.

All analyses were carried out in python. Data and code for reproducing the results of this study are available at https://github.com/systemsmedicine/Tracking_IAV_from_Blood.
