# Supplementary material for: Predicting Influenza A Virus Infection in the Lung from Hematological Data with Machine Learning
Source: mSystems. 2022 Nov 8;7(6):e00459-22. doi: 10.1128/msystems.00459-22 (PMC9765554; doi:10.1128/msystems.00459-22)
Supplement: FIG S5 [file msystems.00459-22-s0005.pdf]

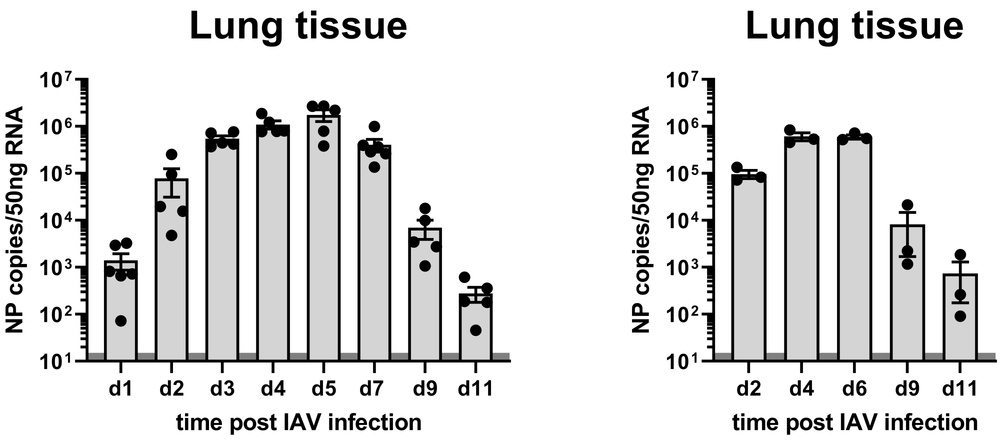

Figure S5. Lung viral load during IAV infection. Wild-type C57BL/6JOlaHsd mice were intranasally inoculated with Influenza A virus (IAV) strain A/PR/8/34 (H1N1) on day 0. At indicated time points, RNA from lung tissue homogenates was extracted and viral burden was assessed by quantifying viral nucleoprotein (NP) copy numbers using quantitative real-time RT-PCR. Data for individual mice and mean±SEM are graphed. Dark grey shades indicate the detection limit. Left panel: Data from first experiment (model setup and training). Right panel: Data from third experiment (model testing and evaluation).
