# Supplementary material for: Predicting Influenza A Virus Infection in the Lung from Hematological Data with Machine Learning
Source: mSystems. 2022 Nov 8;7(6):e00459-22. doi: 10.1128/msystems.00459-22 (PMC9765554; doi:10.1128/msystems.00459-22)
Supplement: FIG S7 [file msystems.00459-22-s0007.pdf]

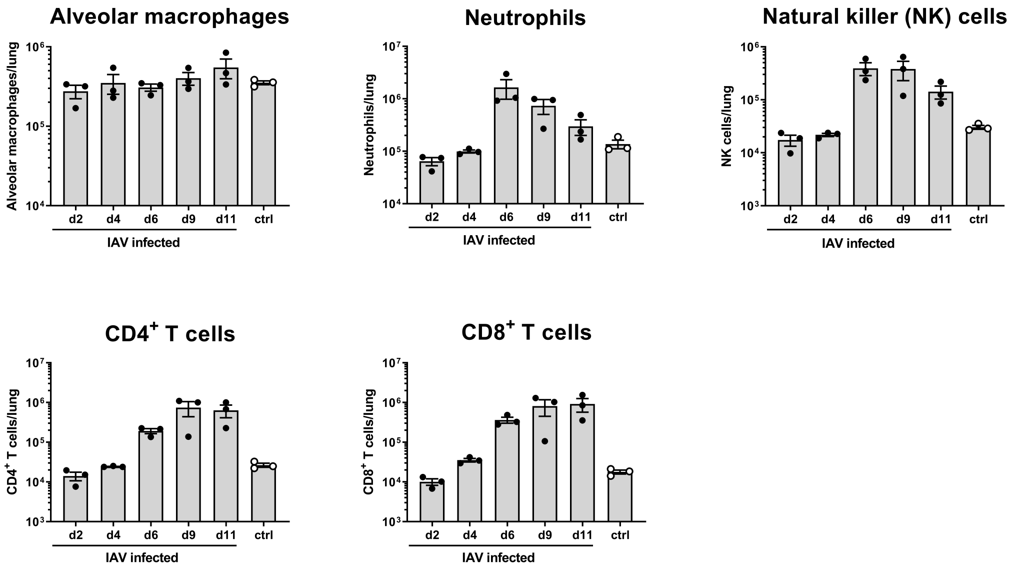

Figure S7. Lung leukocytes during IAV infection (fourth experiment, model validation): Wild-type C57BL/6JOlaHsd mice were intranasally inoculated with Influenza A virus (IAV) strain A/PR/8/34 (H1N1) or treated with PBS (ctrl) on day 0. At indicated time points, lung tissue leukocytes were quantified using flow cytometry. Data for individual mice and mean±SEM are graphed.
