# Supplementary material for: Predicting Influenza A Virus Infection in the Lung from Hematological Data with Machine Learning
Source: mSystems. 2022 Nov 8;7(6):e00459-22. doi: 10.1128/msystems.00459-22 (PMC9765554; doi:10.1128/msystems.00459-22)
Supplement: FIG S9 [file msystems.00459-22-s0009.pdf]

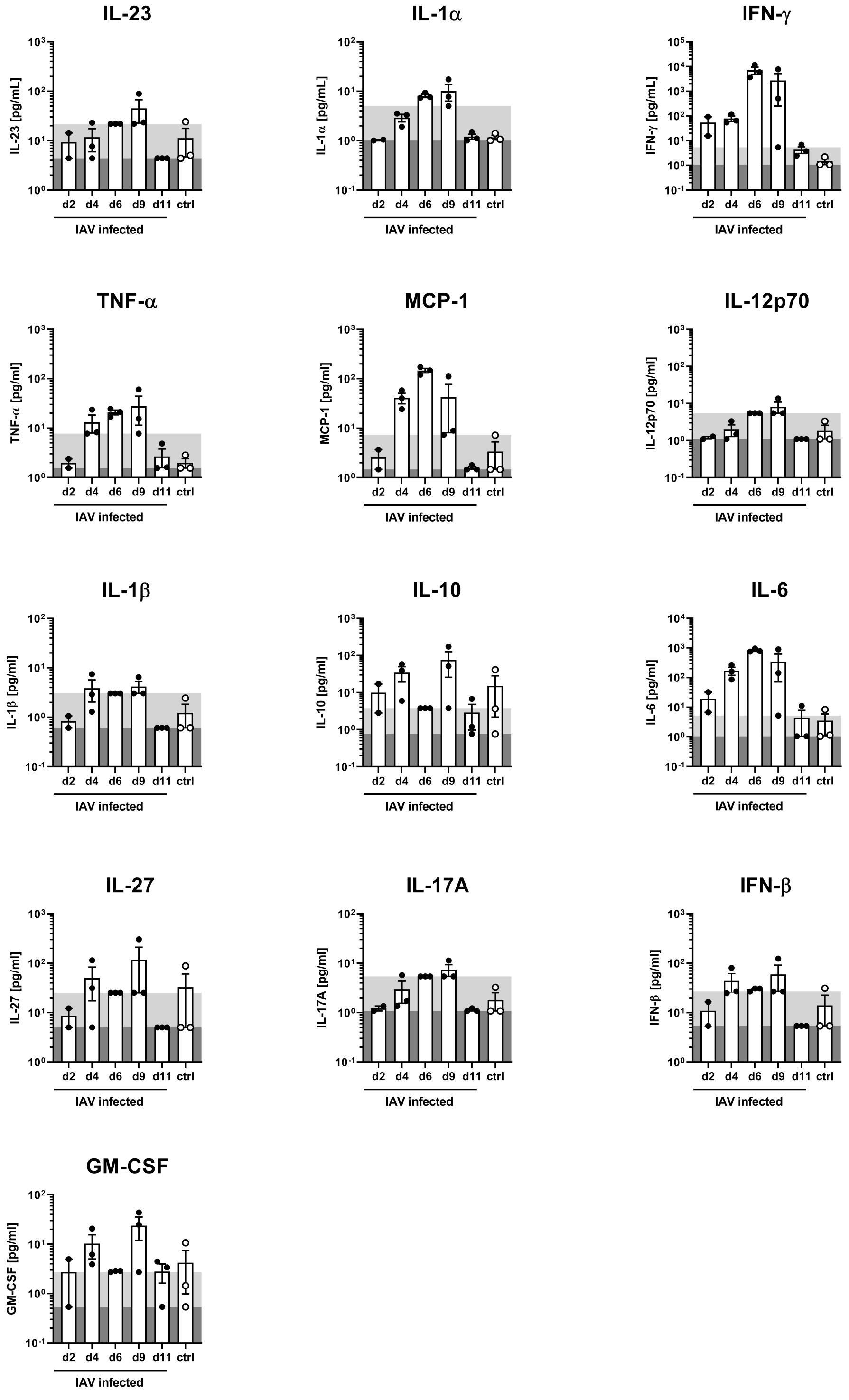

Figure S9. Lung cytokines and chemokines during IAV infection (fourth experiment, model validation). Wild-type C57BL/6JOlaHsd mice were intranasally inoculated with Influenza A virus (IAV) strain A/PR/8/34 (H1N1) or treated with PBS (ctrl) on day 0. At indicated time points, concentrations of cytokines and chemokines in the airways were determined by multiplex analyses. Data for individual mice and mean±SEM are graphed. Dark grey shade indicates the detection limit for samples from ctrl, d2, d4 and d11. Light grey shade indicates detection limit for samples from d6 and d9.
